# Timely Activation of Budding Yeast APC^Cdh1^ Involves Degradation of Its Inhibitor, Acm1, by an Unconventional Proteolytic Mechanism

**DOI:** 10.1371/journal.pone.0103517

**Published:** 2014-07-29

**Authors:** Michael Melesse, Eunyoung Choi, Hana Hall, Michael J. Walsh, M. Ariel Geer, Mark C. Hall

**Affiliations:** 1 Department of Biochemistry, Purdue University, West Lafayette, Indiana, United States of America; 2 Center for Cancer Research, Purdue University, West Lafayette, Indiana, United States of America; Florida State University, United States of America

## Abstract

Regulated proteolysis mediated by the ubiquitin proteasome system is a fundamental and essential feature of the eukaryotic cell division cycle. Most proteins with cell cycle-regulated stability are targeted for degradation by one of two related ubiquitin ligases, the Skp1-cullin-F box protein (SCF) complex or the anaphase-promoting complex (APC). Here we describe an unconventional cell cycle-regulated proteolytic mechanism that acts on the Acm1 protein, an inhibitor of the APC activator Cdh1 in budding yeast. Although Acm1 can be recognized as a substrate by the Cdc20-activated APC (APC^Cdc20^) in anaphase, APC^Cdc20^ is neither necessary nor sufficient for complete Acm1 degradation at the end of mitosis. An APC-independent, but 26S proteasome-dependent, mechanism is sufficient for complete Acm1 clearance from late mitotic and G1 cells. Surprisingly, this mechanism appears distinct from the canonical ubiquitin targeting pathway, exhibiting several features of ubiquitin-independent proteasomal degradation. For example, Acm1 degradation in G1 requires neither lysine residues in Acm1 nor assembly of polyubiquitin chains. Acm1 was stabilized though by conditional inactivation of the ubiquitin activating enzyme Uba1, implying some requirement for the ubiquitin pathway, either direct or indirect. We identified an amino terminal predicted disordered region in Acm1 that contributes to its proteolysis in G1. Although ubiquitin-independent proteasome substrates have been described, Acm1 appears unique in that its sensitivity to this mechanism is strictly cell cycle-regulated via cyclin-dependent kinase (Cdk) phosphorylation. As a result, Acm1 expression is limited to the cell cycle window in which Cdk is active. We provide evidence that failure to eliminate Acm1 impairs activation of APC^Cdh1^ at mitotic exit, justifying its strict regulation by cell cycle-dependent transcription and proteolytic mechanisms. Importantly, our results reveal that strict cell-cycle expression profiles can be established independent of proteolysis mediated by the APC and SCF enzymes.

## Introduction

Proper execution of the eukaryotic cell division cycle depends heavily on ubiquitin-mediated proteolysis, involving the conjugation of polyubiquitin chains to substrate proteins by E3 ubiquitin ligases and their subsequent recognition and degradation by the 26S proteasome [1]. Coupled with transcriptional regulation, proteolysis helps establish cell cycle-dependent protein expression profiles for many key regulators of cell division, contributing to precise control of the initiation and order of cell cycle events [2], [3]. Two large ubiquitin ligase complexes are responsible for the majority of regulated proteolysis during the cell division cycle [2], [4], [5]. One, the Skp1/cullin/F-box protein complex (SCF) is well known for promoting the degradation of G1 cyclins, cyclin-dependent kinase (Cdk) inhibitors, and numerous other substrates, and is thought to be constitutively active. However, recognition of most SCF substrates requires their cell cycle-dependent phosphorylation [6]. The second, the anaphase-promoting complex (APC), or cyclosome, targets the chromosome segregation inhibitor securin, S and M phase cyclins, and many other proteins for degradation during mitosis and G1 [7], [8]. In contrast to SCF, the activity of APC is cell cycle-regulated by several mechanisms including phosphorylation of, and inhibitor binding to, its activator proteins Cdc20 and Cdh1 [9]. Following conjugation of polyubiquitin chains to substrate lysines by SCF and APC, recognition by the 26S proteasome results in their irreversible degradation, and helps drive the cell cycle forward.

In this report, we describe an unconventional proteolytic mechanism, independent of SCF and APC, that helps establish the strict cell cycle expression profile of the APC inhibitor Acm1 in budding yeast. Acm1 was identified several years ago by our lab as a tight binding partner and inhibitor of the APC activator Cdh1 [10], [11]. Acm1 uses substrate-like degron sequences to competitively inhibit substrate binding to Cdh1, making it one of several pseudosubstrate inhibitors of the APC identified in diverse eukaryotes. One important function of Acm1 appears to be ensuring proper positioning of the nucleus along the mother-bud axis prior to nuclear division. Acm1 does this by limiting the premature accumulation of Cdh1 at the bud neck via interaction with its high affinity substrate Hsl1 [12], although the details of how this contributes to proper nuclear orientation remains unclear.

Acm1 expression is very tightly cell cycle-regulated. Acm1 protein is absent from G1 cells, appears around the onset of S phase, and rapidly disappears in late mitosis, after anaphase onset [10], [11], [13]. The *ACM1* promoter is also cell cycle regulated as part of a large collection of genes turned on at the beginning of S phase [14]. Two distinct proteolytic mechanisms have been reported to clear cells of Acm1 at the end of mitosis. First, consistent with the cell cycle profile of Acm1 being reminiscent of APC substrates, Acm1 was shown to be a target of APC^Cdc20^ during anaphase [13]. In other studies, Acm1 was shown to be very sensitive to an APC-independent proteolytic mechanism in G1 [15], [16]. This APC-independent mechanism is inhibited by Cdk phosphorylation on Acm1 such that Acm1 is stable only during the cell cycle window of high Cdk activity. The results presented here provide confirmation of both proteolytic mechanisms but demonstrate that the APC-independent mechanism is both necessary and sufficient for complete elimination of Acm1 during mitotic exit. Interestingly, several lines of evidence suggest that this mechanism is independent of the conventional ubiquitin conjugation pathway, although it is still mediated by the 26S proteasome. This is one of the first examples of a strict cell cycle expression pattern established by proteolytic mechanisms independent of SCF and APC. The existence of two distinct degradation pathways for Acm1 suggests it is critical for yeast cells to relieve Cdh1 inhibition in a timely manner to promote mitotic exit, cytokinesis, and establishment of the ensuing G1 and we provide evidence to support this.

## Results

### APC^Cdc20^ activity is not sufficient for complete Acm1 degradation

**Figure 1:**
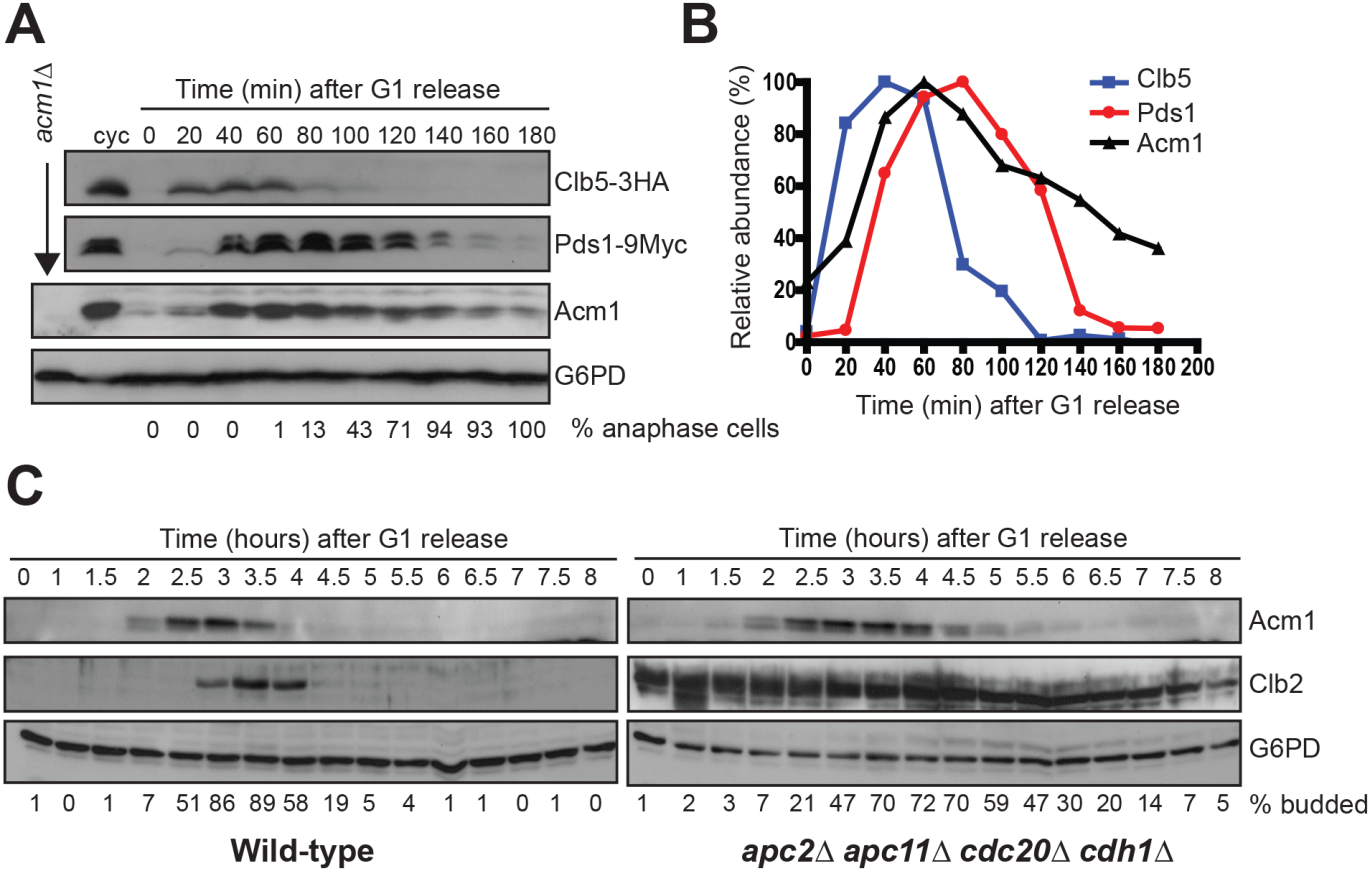
APC^Cdc20^ is neither necessary not sufficient for complete Acm1 degradation at mitotic exit. A) *dbf2-2* cells expressing endogenous chromosomally tagged Clb5-3HA and Pds1-9Myc were released from a G1 arrest at 37°C and the levels of Clb5, Pds1, and Acm1 monitored by immunoblotting over the indicated time period. G6PD is a loading control. Numbers under each lane were obtained by fluorescence microscopic analysis of at least 100 cells at that timepoint stained with DAPI and scored for the presence of 2 segregated DNA masses indicative of the *dbf2-2* late anaphase arrest point. cyc, asynchronous cycling cultures. B) Protein levels were quantified from the immunoblots in panel A. The abundance of each protein was plotted as a percentage of its maximal expression level. C) Extracts from synchronized cultures of yBRT135 (Wild-type) and mutant strain yBRT159 lacking several subunits of APC (*apc2Δ apc11Δ cdc20Δ cdh1Δ pds1Δ clb5Δ SIC1^10x^*) were generously provided by David Toczyski [17], and were probed for Acm1, Clb2, and the loading control G6PD by immunoblotting. The budding index under each lane, taken from [17], is used as an indicator of cell cycle progression.

Acm1 was reported to be effectively eliminated via APC^Cdc20^ prior to mitotic exit [13]. However, we originally identified Acm1 as a Cdh1 binding partner at the late anaphase arrest point of a *cdc15-2* strain [11] and therefore suspected that APC^Cdc20^ might not be sufficient for complete elimination of Acm1. To test this rigorously we had a polyclonal antibody raised against recombinant Acm1 so we could monitor endogenous Acm1 protein without addition of an epitope tag. Synchronized *dbf2-2* cultures were released from α-factor-induced G1 arrest into fresh medium at 37°C so they would arrest in late anaphase, a point where APC^Cdc20^ has been activated and has targeted its substrates for degradation (Figure 1A and 1B). As expected, the levels of the well-characterized Cdc20 substrates Clb5 and Pds1 rapidly dropped as cells reached the arrest point. In contrast, the Acm1 level decreased more slowly and a substantial fraction (>30%) of Acm1 remained after a lengthy arrest.

Next, to definitively test for the presence of an APC-independent proteolytic mechanism acting on endogenous Acm1 protein in late mitosis and G1, we probed for Acm1 in extracts from synchronized cultures of a yeast strain (*apc2Δ apc11Δ cdc20Δ cdh1Δ pds1Δ clb5Δ SIC1^10x^*) engineered to survive in the complete absence of APC activity by deletion of the essential APC substrate genes *CLB5* and *PDS1*, and 10-fold overexpression of the Cdk inhibitor Sic1 [17]. When compared with a control strain containing wild-type APC, Acm1 levels cycled normally (Figure 1C), being absent from G1 cells as previously reported [11]. In contrast, the APC substrate Clb2 was strongly stabilized and present throughout the cell cycle in the absence of APC activity. These results are consistent with previous studies of overexpressed Acm1 stability in G1-arrested cells harboring a conditional APC mutant allele [15], [16]. We conclude that APC^Cdc20^ is not sufficient for complete elimination of Acm1 in late mitosis and that an APC-independent mechanism is also required. In the absence of APC activity this APC-independent mechanism is sufficient for complete Acm1 elimination in late mitosis and G1. We therefore set out to characterize the APC-independent proteolytic mechanism responsible for Acm1 degradation.

### Acm1 is degraded by the 26S proteasome in vivo

**Figure 2:**
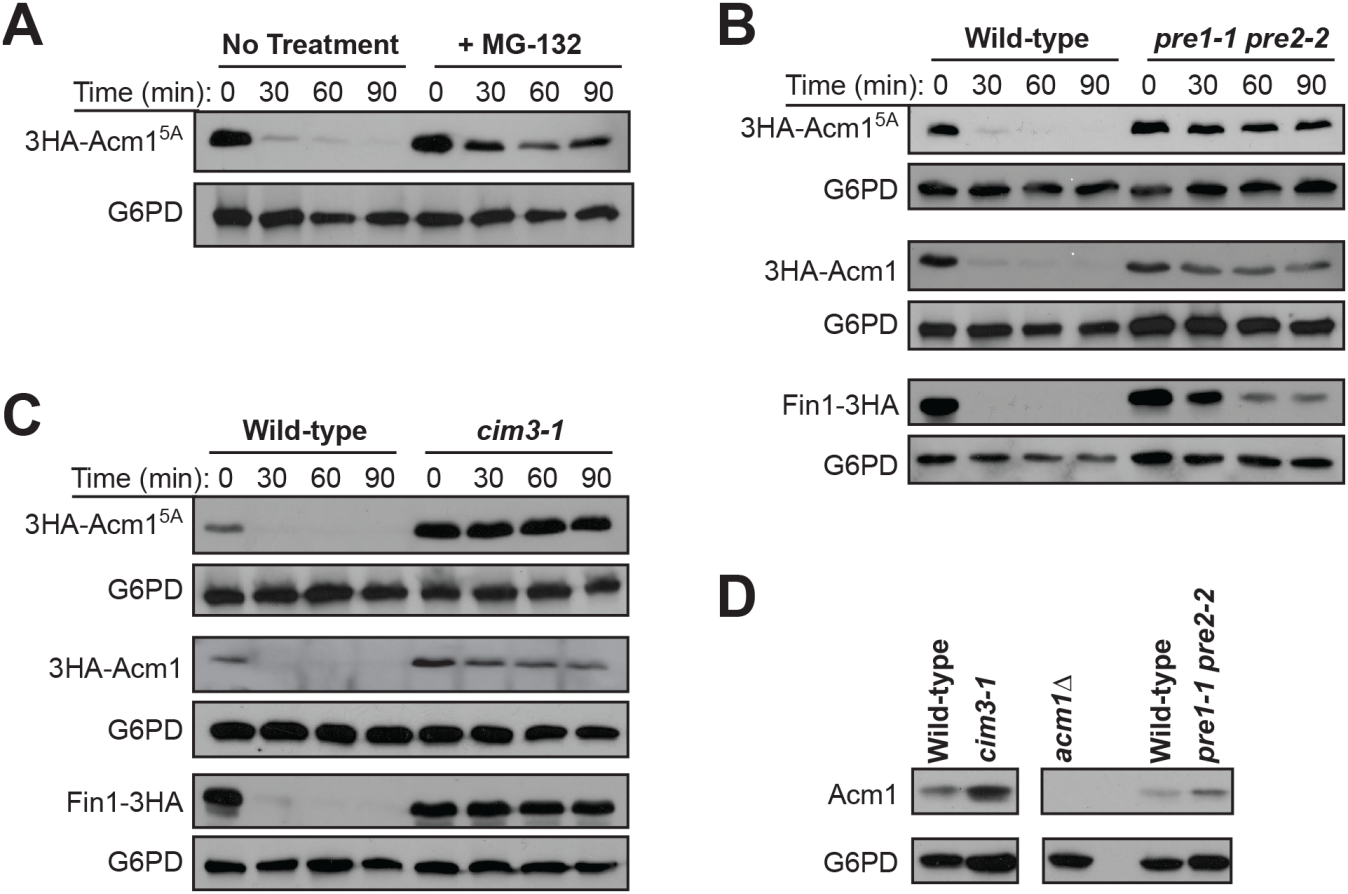
Acm1 degradation requires both the 20S core particle and the 19S regulatory complex of the 26S proteasome. A) Strain YKA407 carrying plasmid pHLP298 expressing 3HA-Acm1^5A^ from the *GAL1* promoter was treated first with galactose to induce 3HA-Acm1^5A^ expression, second with 50 µM MG-132 or a mock treatment, and third with glucose and cycloheximide to terminate expression (Time = 0). The level of 3HA-Acm1^5A^ was then monitored over time by immunoblotting with an HA antibody. G6PD is a loading control. B) and C) The same experiment described in panel A was performed with wild-type (YWO0607 for B, MHY753 for C) or the indicated temperature-sensitive proteasome mutant strains (YWO0612 for B, MHY754 for C) carrying plasmids expressing either 3HA-Acm1^5A^, 3HA-Acm1, or Fin1-3HA from the *GAL1* promoter. Instead of MG-132 treatment, cultures were shifted to 37°C prior to terminating protein expression. For 3HA-Acm1, and Fin1-3HA, cells were arrested first in G1. D) The same strains from panels B and C were grown to exponential phase and shifted to the restrictive temperature to compare the steady-state level of endogenous Acm1 by immunoblotting with an anti-Acm1 antibody. G6PD was used as a loading control.

Proteolysis of Acm1 in G1 can be blocked by the proteasome inhibitor MG-132 [15], suggesting that Acm1 is a substrate of the proteasome. To more rigorously characterize proteasome dependence and rule out the possibility of off-target effects of MG-132 we compared Acm1 stability after MG-132 treatment with stability in a collection of conditional proteasome mutant strains. We used wild-type Acm1, which is unstable only in G1 cells, and an Acm1 mutant lacking Cdk phosphorylation sites (Acm1^5A^), which is unstable throughout the cell cycle [15]. Consistent with our previous results with wild-type Acm1, 3HA-Acm1^5A^ was strongly stabilized after MG-132 treatment in a *GAL1* promoter shutoff/cycloheximide chase assay (Figure 2A). Using the same assay, the stability of both 3HA-Acm1 and 3HA-Acm1^5A^ was measured in G1-arrested *pre1-1 pre2-2* cells harboring temperature-sensitive mutations in the β4 and β5 subunits of the 20S proteasome core particle [18]. Both 3HA-Acm1 and 3HA-Acm1^5A^, as well as the control APC substrate Fin1-3HA were highly unstable in wild type G1 cells but were strongly stabilized in the *pre1-1 pre2-2* mutant strain (Figure 2B). These results confirm that Acm1 is degraded by the proteasome in G1. We also tested Acm1 stability in a proteasome mutant strain, *cim3-1*, with a temperature-sensitive defect in the Rpt6 ATPase subunit of the 19S regulatory particle [19], which is primarily responsible for recognition and processing of poly-ubiquitinated proteins. The *cim3-1* strain shows defective proteolysis of ubiquitinated substrates at restrictive temperature [19]. 3HA-Acm1, 3HA-Acm1^5A^, and Fin1-3HA were all strongly stabilized in G1-arrested *cim3-1* cells (Figure 2C). To ensure this was not an artifact of Acm1 overexpression from the *GAL1* promoter we also monitored the level of endogenous Acm1 in wild-type and conditional proteasome mutant strains at the restrictive temperature. The steady-state level of Acm1 was increased upon proteasome inactivation in both *cim3-1* and *pre1-1 pre2-2* cells (Figure 2D). Collectively, these results demonstrate that Acm1 proteolysis requires activity of the 26S proteasome, not just the 20S core particle, and suggested that Acm1 is likely targeted to the proteasome via polyubiquitination. We therefore set out to identify components of the ubiquitin system required for the APC-independent Acm1 proteolysis.

### Acm1 proteolysis requires a functional ubiquitin conjugation system

**Figure 3:**
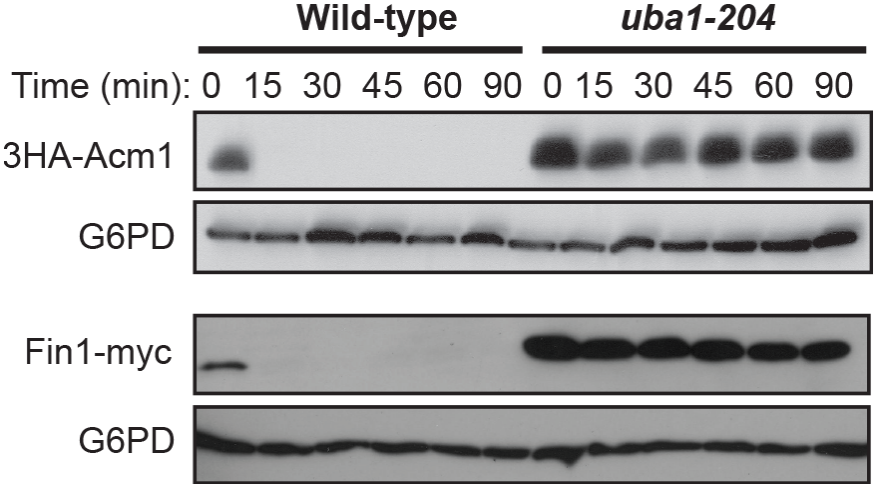
Acm1 proteolysis requires a functional ubiquitin conjugation pathway. Expression of 3HA-Acm1 or Fin1-myc was induced with galactose in G1-arrested wild-type (RJD3268) or *uba1-204* (RJD3269) cells at room temperature. Culture temperature was increased to 37°C prior to addition of glucose and cycloheximide. Cells were harvested at the indicated times after terminating expression and protein levels monitored by immunoblotting with anti-HA or anti-myc antibodies. G6PD is a loading control.

To test if Acm1 proteolysis is generally dependent on the ubiquitin conjugation system, we measured its stability in a strain harboring a conditional mutation, *uba1-204,* in the sole E1 ubiquitin activating enzyme in budding yeast [20]. A pulse of 3HA-Acm1 or Fin1-myc expression was induced from the *GAL1* promoter in G1-arrested wild-type and *uba1-ts* cells after shift to the non-permissive temperature of 37°C (Figure 3). Both 3HA-Acm1 and the control protein Fin1-myc were strongly stabilized in *uba1-ts* cells compared to isogenic wild-type cells. Similar, although less dramatic, results were obtained using a second conditional E1 allele, *uba1-ts* ([21]; data not shown). We conclude that the APC-independent G1 degradation of Acm1 is dependent on a functional ubiquitin conjugation system.

### Acm1 proteolysis does not require individual E3 ligases or E2 conjugating enzymes

To identify the proteins directly responsible for Acm1 ubiquitination and proteolysis, we systematically screened a collection of yeast strains individually lacking non-essential yeast E2 ubiquitin conjugating enzymes and E3 ubiquitin ligases (Table S1) for effects on Acm1 stability. We used the Acm1^5A^ mutant because it is constitutively unstable and does not require cell cycle arrest in G1 to monitor effects on stability. First, steady-state levels of 3HA-Acm1^5A^ expressed from the natural *ACM1* promoter in exponentially growing E2 and E3 mutant cultures were measured by immunoblotting and compared to the level of wild-type 3HA-Acm1 in the parent strain. Surprisingly, the level of Acm1^5A^ fluctuated widely in the mutant strains, and in several cases was significantly elevated, suggesting that Acm1 stability might be increased in the absence of these ubiquitin system components (Figure S1). However, subsequent direct testing of stability using our inducible promoter and cycloheximide chase system failed to reveal any strong effects of these gene deletions on Acm1 half-life (Figure S2). We suspect that the differences in steady-state expression from the natural promoter were likely due to effects on transcription or cell cycle distribution since the *ACM1* promoter is strictly cell cycle regulated [14]. Consistent with this, analysis of wild-type Acm1 and Acm1^5A^ levels in G1-arrested cultures of these deletion strains cells failed to reveal evidence of stabilization (data not shown).

To avoid problems with expression from the highly regulated *ACM1* promoter we re-screened the entire collection of E2 and E3 deletion strains using the *GAL1* promoter stability assay and directly monitored stability of untagged Acm1^5A^ with our Acm1 antibody. We also added strains lacking genes encoding predicted RING domains without known E3 activity and other recently verified E3 ligases (Table S1). Since inhibition of either the proteasome or Uba1 resulted in strongly stabilized Acm1 over the course of at least one hour in this assay, we compared the level of Acm1 at 0 and 60 minutes in each strain after addition of glucose and cycloheximide. In 47 known or putative E3 deletion strains and 10 E2 deletion strains we did not find a single case where Acm1 was stabilized comparable to MG-132 addition (Figure S3 and data not shown). We also found no effect of the essential E3 enzymes Rsp5 and Prp19 on Acm1 stability using strains from the tetracycline-repressible essential gene library (data not shown). The essential E3 SCF was tested previously with negative results [16] and we confirmed these results using conditional *cdc4* and *cdc53* alleles (data not shown). We conclude that Acm1 proteolysis by the APC-independent mechanism does not require any single E2–E3 modules, although we cannot rule out the possibility that multiple redundant E3 enzymes are capable of promoting Acm1 degradation or that an unknown E3 exists that was not tested.

### Acm1 proteolysis does not require assembly of ubiquitin chains

**Figure 4:**
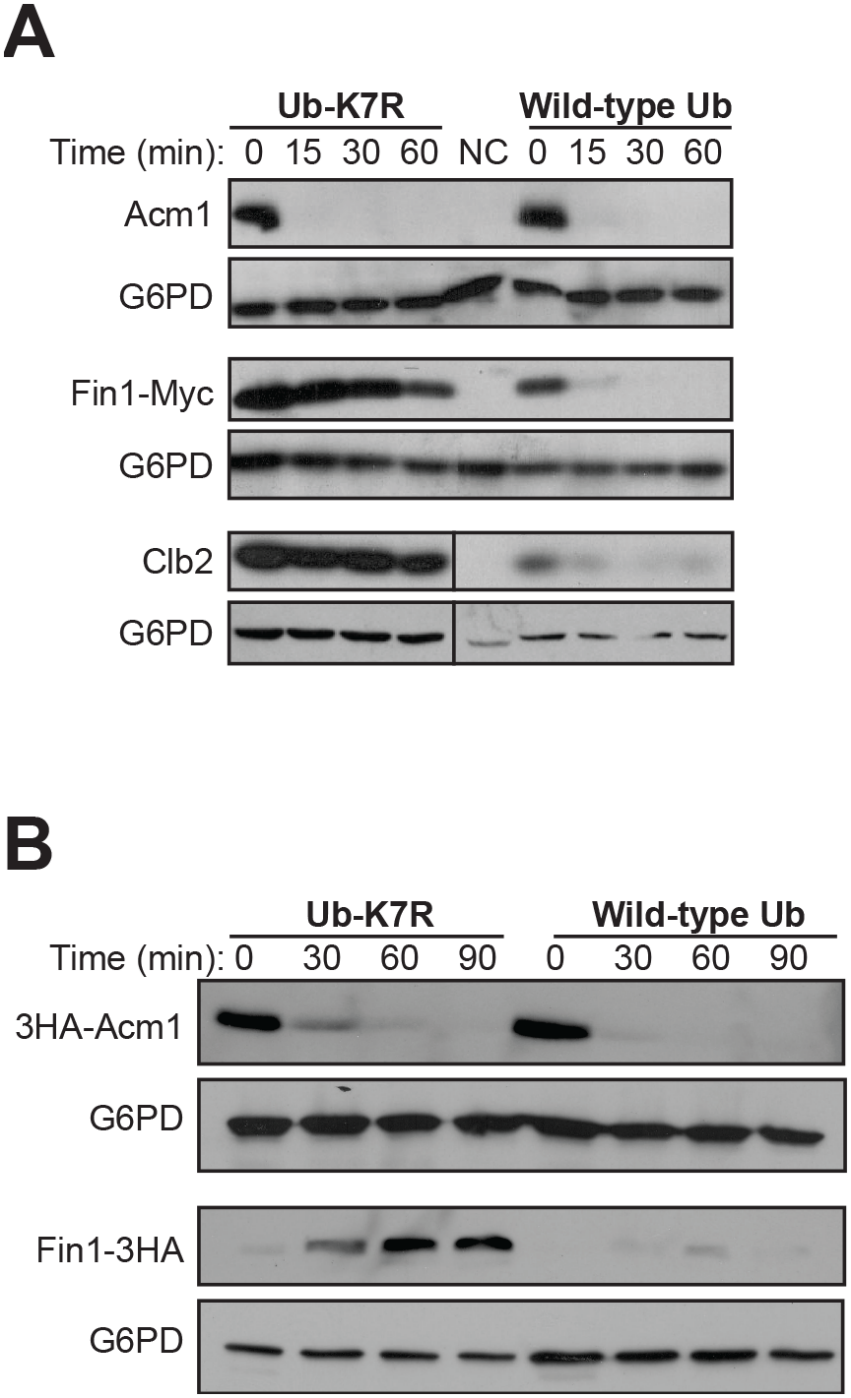
Acm1 proteolysis does not require assembly of polyubiquitin chains. A) YKA247 cells containing *P_GAL1_*-driven plasmids pHLP391 (for Acm1), pESCW-Fin1-Myc, or pHLP309 (for Clb2) and *P_CUP1_*-driven ubiquitin (Ub) overexpression plasmids LHP306 (for Ub-K7R mutant) or LHP308 (for wild-type Ub) were grown to early-exponential phase. Cells were arrested at G1 before expression of wild-type or mutant Ub was induced with 100 µM CuSO_4_ and Acm1, Fin1-myc, and Clb2 with galactose. Stability of Acm1, Fin1-Myc, and Clb2 were monitored with anti-Acm1, anti-Myc, and anti-Clb2 antibodies, respectively. G6PD is a loading control. NC, negative control without galactose induction. B) The same experiment described in panel A was performed in *doa4Δ* cells to limit the abundance of endogenous ubiquitin, and only glucose was used to terminate expression, allowing continuous synthesis of mutant or wild-type Ub. In this experiment pHLP212 was used to express 3HA-Acm1, which was detected with an anti-HA antibody and Fin1-3HA was used as a control.

In parallel with the *uba1-204* experiment described above, we analyzed Acm1 stability after overexpressing a mutant ubiquitin in which all lysines have been replaced with arginine (Ub-K7R, a gift from L. Hicke, Northwestern University). This mutant blocks poly-ubiquitin chain extension when conjugated to a protein [22] and thereby stabilizes ubiquitin proteasome substrates. The stabilities of Acm1 and the APC substrates Fin1 and Clb2 were monitored in G1-arrested cells overexpressing either mutant or wild-type ubiquitin. As expected, Fin1 and Clb2 were highly stabilized by overexpression of Ub-K7R (Figure 4A). Their steady-state levels were also noticeably higher at time 0 in the presence of Ub-K7R compared to wild-type ubiquitin. In contrast, the stability of Acm1 was unaffected by the overexpressed chain-terminating Ub-K7R and the steady state level of Acm1 was similar at time 0 in both strains. Similar results were observed with 3HA-Acm1 in a *doa4Δ* strain, which has a defect in processing of ubiquitin precursors and therefore a lower level of endogenous ubiquitin for Ub-K7R to compete with (Figure 4B). In this case, we terminated transcription from the *GAL1* promoter with glucose but did not add cycloheximide, allowing continuous synthesis and accumulation of Ub-K7R and wild-type ubiquitin. Surprisingly, these experiments reveal that Acm1 proteolysis in G1 shows no apparent dependence on assembly of poly-ubiquitin chains, a general requirement for ubiquitin-mediated proteolysis.

### Ubiquitin conjugation sites on Acm1 are not required for its proteolysis

**Figure 5:**
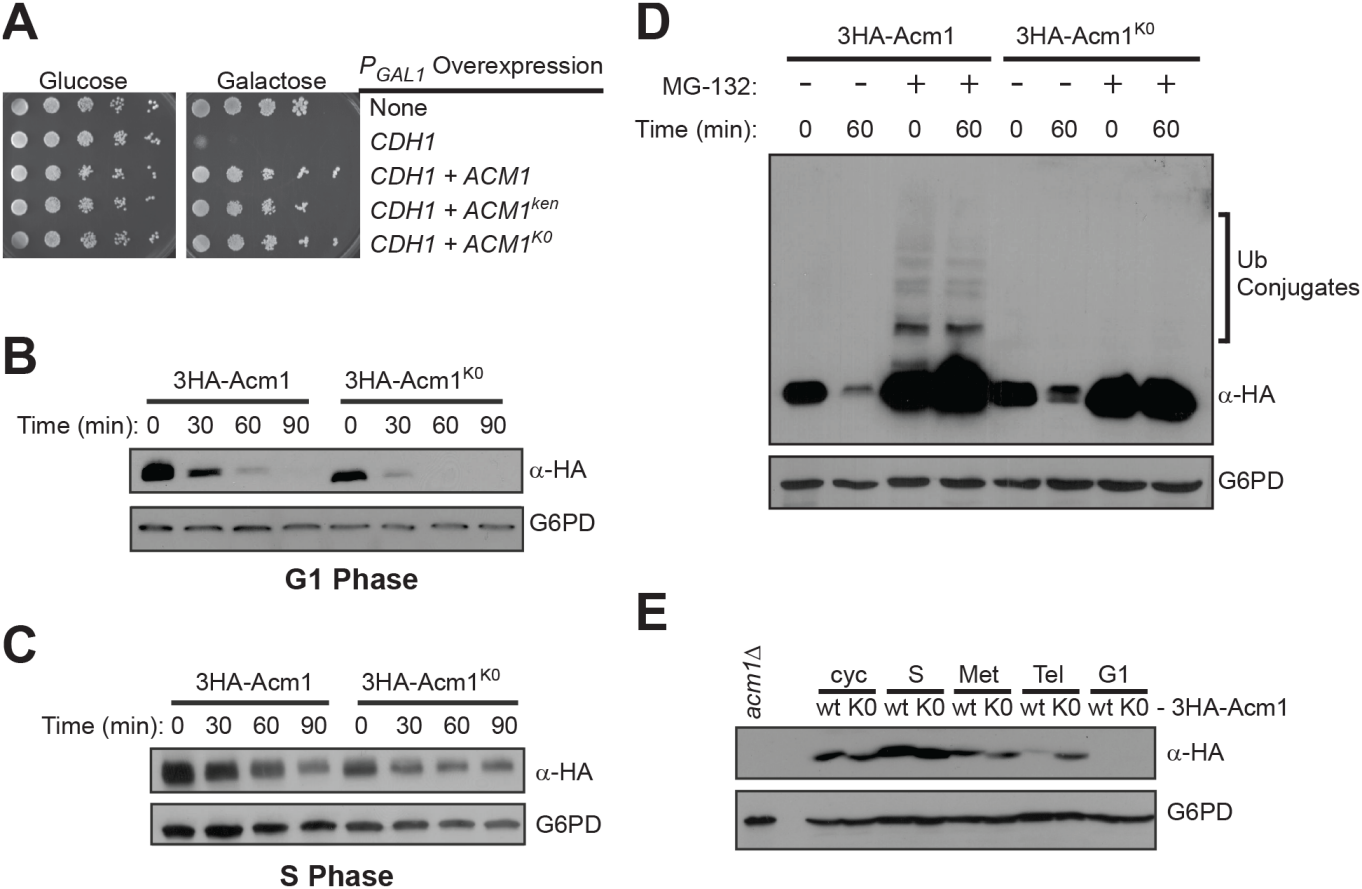
Acm1 proteolysis does not require ubiquitin acceptor sites on Acm1. A) YKA247 cells carrying *P_GAL1_* expression plasmids for 3FLAG-Cdh1, 3HA-Acm1, HA-Acm1-ken, and 3HA-Acm1^K0^ in the indicated combinations were grown until mid-exponential phase. 10-fold serial dilutions were spotted on selective media containing either glucose or galactose. Plates were incubated at 30°C for 2–3 days. B) and C) YKA247 cells carrying *P_GAL1_* expression plasmids for 3HA-Acm1 (pHLP117) or lysine-less 3HA-Acm1^K0^ mutant (pHLP330) were grown in YP-raffinose to early exponential phase. Cells were arrested at G1 (panel B) or S phase (panel C). Stability of 3HA-Acm1 and 3HA-Acm1^K0^ was monitored over the indicated time period by immunoblotting with anti-HA antibody. G6PDH is a loading control. D) Same as panels B and C, except *pdr5Δ* cells were used and stability was monitored in the presence and absence of MG-132 as indicated. Longer immunoblot exposures were obtained for detection of ubiquitin (Ub) conjugates. E) *cdc15-2* cells carrying centromeric plasmids expressing either wild-type 3HA-Acm1 or 3HA-Acm1^K0^ from the natural *ACM1* promoter were arrested at the indicated cell cycle stages as described in Materials and Methods. The level of each protein was then compared by anti-HA immunoblotting with G6PD as a loading control.

To independently test the dependence of Acm1 proteolysis on ubiquitination, we constructed a mutant *ACM1* allele in which all 20 lysine codons were replaced with arginine codons. The mutant protein, 3HA-Acm1^K0^, lacks all internal sites for ubiquitin conjugation and we added a 3HA epitope tag lacking lysines to block the native N-terminus as a potential ubiquitin conjugation site. Despite the extensive mutagenesis, 3HA-Acm1^K0^ is fully functional as a Cdh1 inhibitor *in vivo* (Figure 5A) because 3HA-Acm1^K0^ overexpression suppressed the toxic effect of Cdh1 overexpression like wild-type 3HA-Acm1 [10], [11], [16], [23]. Thus, the mutant is biologically functional and does not suffer from global misfolding or severe structural differences compared to wild-type Acm1. We compared the stability of galactose-induced pulses of 3HA-Acm1^K0^ and 3HA-Acm1 in α-factor arrested G1 cells (Figure 5B). The absence of lysines had no effect on the stability of Acm1 (Figure 5B), suggesting that ubiquitin conjugation to Acm1 is not required for its proteolysis. Acm1 stability is dramatically increased by cyclin-dependent kinase phosphorylation [13], [15], [16] and as a result, Acm1 is stable in S and M phase cells. To determine if the Acm1^K0^ mutant is still regulated by phosphorylation like wild-type Acm1, we repeated the stability assay in S phase cells. Both wild-type 3HA-Acm1 and 3HA-Acm1^K0^ were comparably stable in hydroxyurea-arrested S phase cells compared to G1 (Figure 5C), suggesting that the mutagenesis did not perturb normal phospho-regulation of Acm1 stability.

Since in rare cases proteins can undergo ubiquitination at non-lysine sites, we next directly tested for ubiquitin conjugates on Acm1 in G1-arrested cells. Wild-type 3HA-Acm1 and 3HA-Acm1^K0^ were compared in our stability assay with and without proteasome inhibition. Consistent with results presented above, both proteins were highly unstable in the absence of MG-132 but strongly stabilized in the presence of MG-132 (Figure 5D). Acm1 has been proposed to undergo weak ubiquitination and subsequent degradation mediated by APC^Cdh1^, the enzyme it inhibits [13]. Consistent with this, we detected ubiquitin conjugates on wild-type 3HA-Acm1 in the presence of MG-132 (Figure 5D). Under identical conditions, ubiquitin conjugates were undetectable on 3HA-Acm1^K0^. Since these proteins have indistinguishable half-lives in G1 cells, the ubiquitin conjugates detected on wild-type Acm1 are not likely to contribute significantly to its proteolysis under normal conditions. Importantly, these results argue that Acm1^K0^ is not targeted to the proteasome via unconventional ubiquitin linkages to non-lysine amino acids, or to the N-terminus.

Finally, we compared the levels of 3HA-Acm1 and 3HA-Acm1^K0^ expressed from the natural *ACM1* promoter as a function of cell cycle stage in *cdc15-2* cells (Figure 5E). Cells were arrested in G1 with α-factor, S with hydroxyurea, early M with nocodazole, and late M by temperature shift to 37°C and Acm1 levels compared by immunoblotting. In cycling cells and S and early M arrested cells the abundance of 3HA-Acm1 and 3HA-Acm1^K0^ was equivalent. In late M arrested cells, the abundance of 3HA-Acm1^K0^ was higher than 3HA-Acm1 and was equivalent to the level in early M cells, consistent with the anaphase ubiquitin-dependent proteolytic mechanism mediated by APC^Cdc20^ [13]. In support of our results from Figure 1, a portion of the wild-type 3HA-Acm1 was still present in these late anaphase cells, demonstrating that Acm1 is not completely eliminated by APC^Cdc20^. Importantly, 3HA-Acm1^K0^ was undetected in G1 cells, similar to wild-type 3HA-Acm1. This strongly implies that the APC-independent proteolytic mechanism that clears Acm1 completely during mitotic exit and G1 does not involve conjugation of ubiquitin chains to Acm1.

### An N-terminal putative disordered region of Acm1 contributes to APC-independent degradation

**Figure 6:**
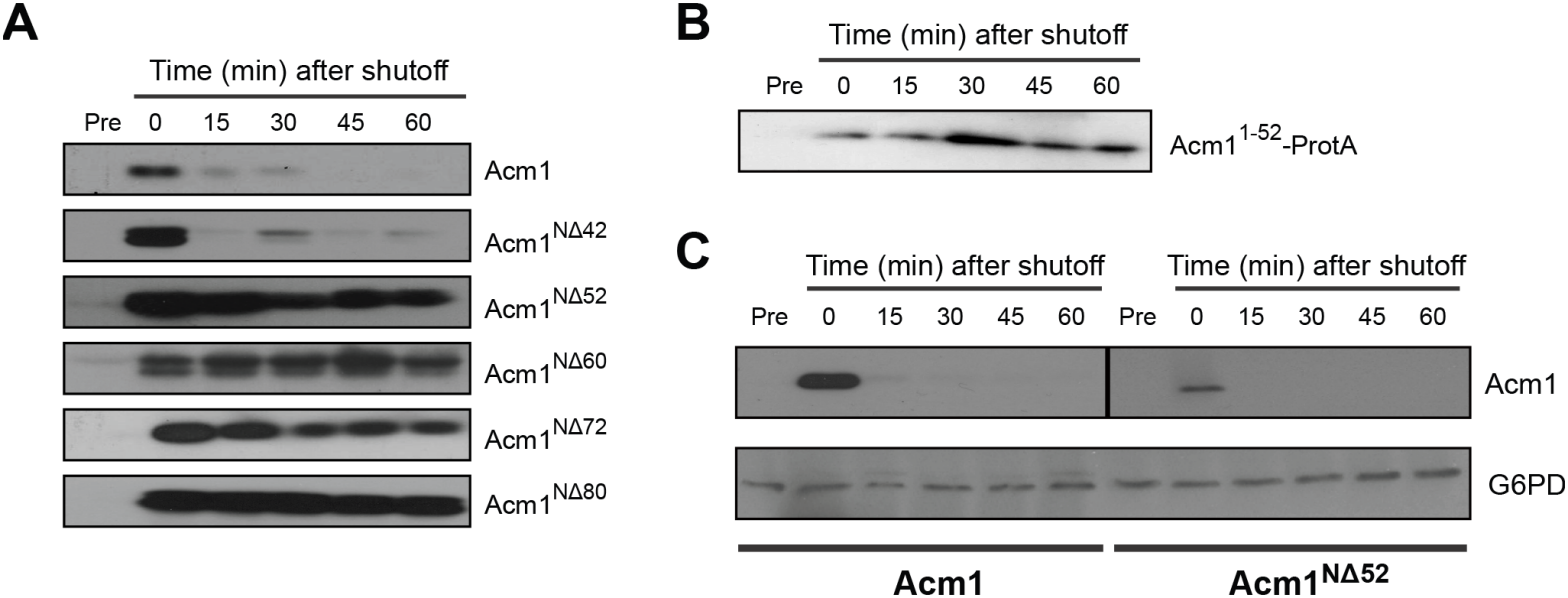
Proteolysis of an Acm1-ProtA fusion protein in G1 requires the N-terminal 52 amino acids of Acm1. A) YKA247 cells transformed with *P_GAL1_* constructs expressing *ACM1*, *acm1^NΔ42^*, *acm1^NΔ52^*, *acm1^NΔ60^*, *acm1^NΔ72^* or *acm1^NΔ80^* fused to the ZZ domain of Protein A were arrested in G1 and protein stability assayed over time by immunoblotting with anti-Protein A antibody. B) The same assay as panel A with cells expressing Acm1 amino acids 1–52 fused to Protein A. C) The stability of Acm1 and Acm1^NΔ52^ without the Protein A fusion were compared using the same assay as in panel A, but with anti-Acm1 antibody for immunoblot detection. G6PD is a loading control. G6PD loading controls were performed for all blots in panels A and B as well (not shown).

To probe the biological significance of Acm1 degradation we needed a stable Acm1 mutant. We therefore sought truncated Acm1 variants that exhibited increased half-life in our G1 stability assay. We fused the ZZ domain of Protein A (ProtA) to the Acm1 C-terminus (for equivalent immunoblot detection of all Acm1 constructs). Full length Acm1-ProtA was highly unstable. Removal of 42 amino acids or less had little effect on the rate of Acm1-ProtA degradation (Fig. 6A). Strikingly, removal of the 52 N-terminal amino acids strongly stabilized Acm1-ProtA in this assay and longer truncations from the N-terminus were also stable.

Although clearly required for efficient Acm1 degradation in G1, the first 52 amino acids of Acm1 were not sufficient to destabilize Protein A when fused to its N-terminus (Figure 6B). This suggested that other regions of Acm1 might be important as well. We found that the Acm1^NΔ52^ protein was unstable in the absence of the C-terminal Protein A fusion (Figure 6C). Thus, stabilization of Acm1 in G1 required both loss of the amino terminus and modification of the C-terminus. The N-terminal region of Acm1 is predicted to be highly disordered using several secondary structure prediction algorithms (data not shown). The proteasome can directly recognize some disordered proteins and catalyze their proteolysis in the absence of ubiquitin conjugation. We purified active 20S and 26S proteasomes from yeast but found no evidence that a variety of recombinant Acm1 proteins purified from *E. coli* could be directly recognized and degraded (Figure S4).

### Acm1^NΔ52^ is still cleared from cells at mitotic exit

**Figure 7:**
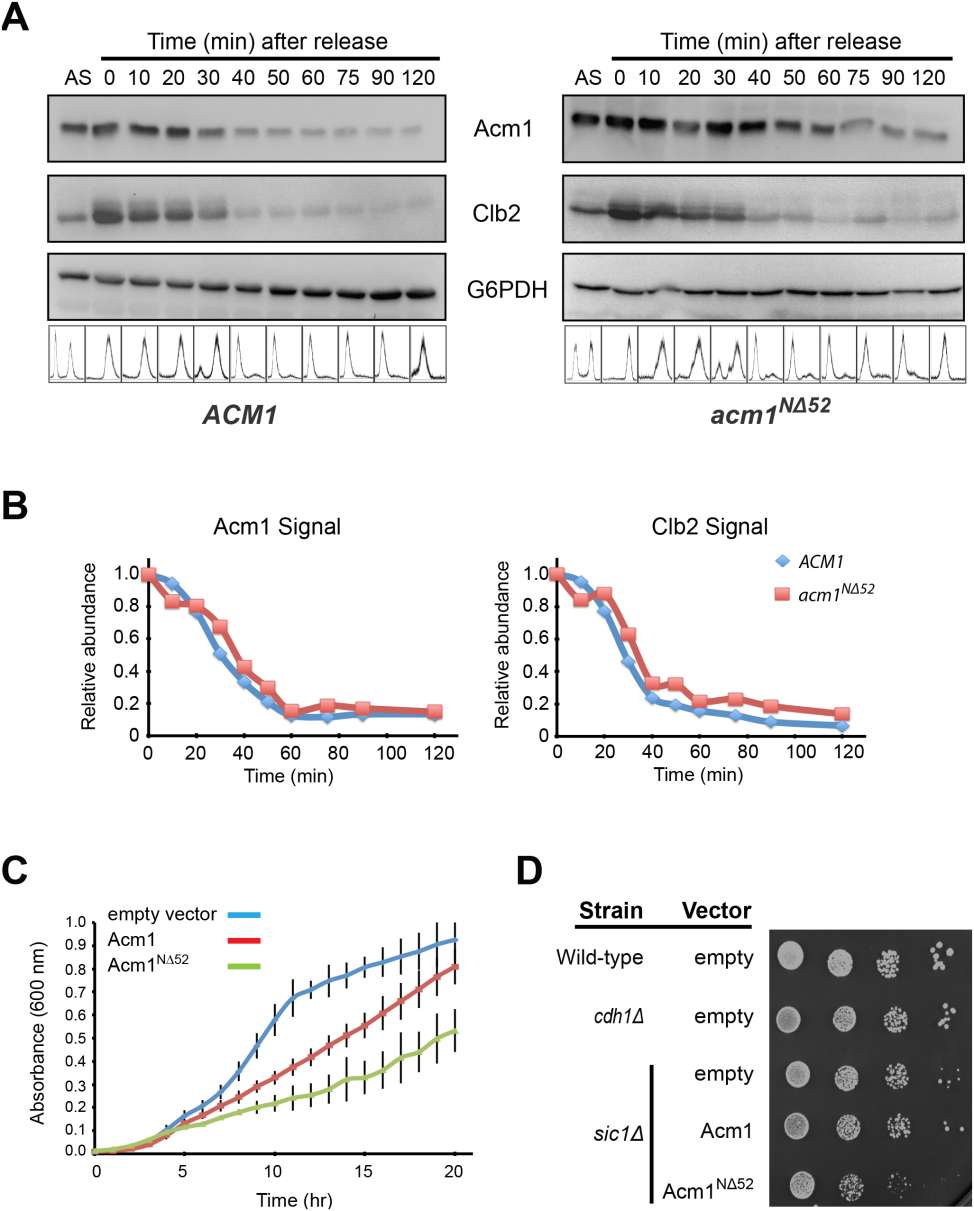
Acm1^NΔ52^ is still cleared at mitotic exit, but constitutive expression impairs growth of *sic1Δ* cells. A) YKA859 cells carrying either pHLP117 (for expression of 3HA-Acm1) or pHLP505 (for expression of Acm1^NΔ52^-ProtA) were arrested at metaphase by methionine repression of *P_MET3_-CDC20* and then released in the absence of methionine. Cells were collected at regular intervals and analyzed by immunoblotting using antibodies against Acm1, Clb2, or G6PD (loading control). B) Quantitation of chemiluminescent immunoblots from panel A. Data are the average of 4 independent experiments. C) Growth of *sic1Δ* cells transformed with either an empty vector, pHLP361 (*P_ADH_*-*Acm1-ProtA*) or pHLP363 (*P_ADH_*-*acm1^NΔ52^-ProtA*) were compared using a plate reader to measure absorbance at 600 nm. Data are the average of three independent experiments with standard deviation error bars. D) Serial 10-fold dilutions of the strains from panel C as well as isogenic wild-type and *cdh1Δ* strains were spotted and grown on selective agar plates.

We next used the stabilized Acm1^NΔ52^ protein to study the biological significance of Acm1 proteolysis at mitotic exit. It is important to note that the N-terminus of Acm1 also contains the Cdc20-specific D-box responsible for Acm1 recognition by APC^Cdc20^ [13]. Thus, the Acm1^NΔ52^ protein should be resistant to both known proteolytic mechanisms. We predicted that failure to degrade Acm1 would prevent or delay activation of APC^Cdh1^. To test this, strains harboring a *P_MET3_-CDC20* allele and expressing either Acm1 or Acm1^NΔ52^-ProtA from the *ACM1* promoter were blocked at metaphase by methionine addition and then released in the absence of methionine to undergo synchronous mitotic exit. The levels of Acm1 and the Cdh1 substrate Clb2 were monitored over time by immunoblotting (Figure 7A and 7B). Surprisingly, there was little difference in the decay profiles of Acm1 and Acm1^NΔ52^-ProtA. Moreover, we did not observe significant differences in degradation of Clb2. Similar results were observed with another Cdh1 target, Kip1 (data not shown). Thus, although Acm1^NΔ52^-ProtA is highly stabilized in G1-arrested cells, it is still effectively cleared by cells during mitotic exit. Possible explanations for this are discussed below.

### Constitutive expression of stabilized Acm1^NΔ52^ impairs growth of sic1Δ cells

Since the *ACM1* promoter is cell cycle-regulated, loss of Acm1^NΔ52^ as cells exit mitosis could be in part due to termination of *ACM1* transcription. To test for defects in APC^Cdh1^ function in cells expressing stabilized Acm1^NΔ52^ we expressed *ACM1* and *acm1^NΔ52^* alleles from the constitutive *ADH* promoter in a *sic1Δ* background. Either Sic1 or Cdh1 is sufficient for Cdk inactivation and mitotic exit in budding yeast, however loss of both is lethal. Therefore, activation of Cdh1 becomes critically important in *sic1Δ* cells. We observed a significant growth delay in *sic1Δ* cells constitutively expressing Acm1^NΔ52^ compared to wild-type Acm1, both in liquid culture and on agar plates (Figure 7C and 7D). This experiment supports the idea that clearance of Acm1 from cells is required for timely and full activation of APC^Cdh1^ at mitotic exit and in G1.

## Discussion

### Acm1 may be a novel ubiquitin-independent proteasome substrate

The 26S proteasome is a large ATP-dependent protease complex consisting of a 20S core particle with a 19S regulatory particle at both ends [24], [25]. It is the primary enzymatic activity responsible for protein turnover in eukaryotic cells [26]. Although the vast majority of known proteasome substrates require ubiquitination for proteolysis, proteins can also be recognized by the proteasome without ubiquitin conjugation [27], [28], [29]. The 20S core particle alone, which is likely the predominant form of the proteasome *in vivo,* can recognize mis-folded or damaged proteins directly and catalyze their proteolysis independent of ubiquitin conjugation and ATP hydrolysis. In addition, the 26S holoenzyme can also recognize some proteins independent of ubiquitin. One recent study estimated that 20% of human proteins are subject to proteolysis by the proteasome independent of ubiquitin conjugation [30]. However the extent to which any of this proteolysis is regulated is unclear and ubiquitin-independent proteolysis is thought to largely constitute a basal protein turnover pathway and/or a route to eliminate damaged or mis-folded proteins [28], [31].

The best-characterized ubiquitin-independent substrate of the 26S proteasome is ornithine decarboxylase (ODC), which catalyzes the rate-limiting first step in polyamine biosynthesis [32], [33]. ODC is targeted to the proteasome by a protein, termed antizyme, in response to high polyamine levels. This regulated ubiquitin-independent proteolysis serves to maintain polyamine homeostasis in cells [34]. Antizyme binding to ODC results in a conformational change that exposes a C-terminal degron in ODC recognized by the 26S proteasome [35], [36]. Antizyme itself also enhances recognition of ODC by the proteasome. The C-terminal tail of ODC may act as a structural ubiquitin mimic since it competes with ubiquitin chains for binding to the proteasome [36]. Interestingly, antizyme has also been linked to the ubiquitin-independent proteasomal degradation of other proteins, including the cell cycle regulators cyclin D1 [37] and Aurora A kinase [38]. Other proteins involved in aspects of cell cycle control with reported ubiquitin-independent proteolytic mechanisms include p53 [39], p21^Cip1^ [40], [41], [42], [43], and Rb [44], [45]. These proteins are also destroyed by the conventional ubiquitin-proteasome pathway and it is often difficult to dissect the relative physiological contributions of ubiquitin-dependent and independent mechanisms. In addition, for all of these cases it is unclear if, and how, ubiquitin-independent proteolytic mechanisms are regulated during cell division. One possible exception to this may be human c-Fos, a proto-oncoprotein that functions as part of the AP-1 transcription factor complex involved in control of cell proliferation and other processes. c-Fos is sensitive to ubiquitin-independent proteolysis and one report suggests that phosphorylation of a C-terminal degron sequence by MAP kinases specifically at the G0/G1 transition inhibits this mechanism [46]. The importance of this mechanism and its relevance in cycling cells is unclear, but it is reminiscent of what we observe with Acm1, which is also stabilized at the G1/S transition by phospho-dependent inhibition of seemingly ubiquitin-independent proteolysis.

Several features of Acm1’s proteolytic mechanism reported here are reminiscent of ubiquitin-independent proteolysis. First, Acm1 degradation is insensitive to inhibition of ubiquitin chain assembly (Figure 4). Second, Acm1 degradation does not require acceptor lysines and does not appear to involve direct ubiquitin conjugation (Figure 5). Third, no individual E3 ligase or E2 conjugating enzyme is required for Acm1 proteolysis. The one notable exception is that Acm1 proteolysis does require a functional E1, suggesting that the ubiquitin conjugation system is at least indirectly required. Thus, Acm1 meets most, but not all, criteria for an ubiquitin-independent proteasome substrate [28].

### How is Acm1 recognized by the proteasome and what is the role of the Acm1 N-terminus?

Additional work will be required to illuminate the biochemical mechanism by which Acm1 is specifically targeted to the proteasome in its dephosphorylated state. Many ubiquitin-independent proteasomal targets are thought to possess unstructured regions that mediate their direct recognition by the proteasome, much like a mis-folded protein [28], [47]. The N-terminus of Acm1 is predicted to be largely disordered (our unpublished observations) and contributes to its APC-independent proteolysis (Figure 6). Phosphorylation at Cdk sites in and around the Acm1 N-terminus may promote a more structured conformation that prevents degradation. Alternatively, Acm1 may interact with a proteasome targeting factor analogous to the ODC-antizyme interaction that has high affinity for dephosphorylated Acm1 but not for phosphorylated Acm1. This factor could itself be regulated by ubiquitination, thereby explaining the E1 result and general requirement for the ubiquitin system. The E1 requirement could also reflect a general role for ubiquitinated proteins in activating the 26S proteasome, a phenomenon that has some recent support [48]. Another possibility is that the Acm1 N- and/or C-terminus contain sequences that are directly recognized by a component of the proteasome. Such a sequence could act by mimicking ubiquitin as proposed for ODC, or interact with a completely distinct proteasome structure.

Despite the strong stabilization of Acm1^NΔ52^-ProtA in our *GAL1* promoter stability assay (Figure 6, half-life >1 hour), it was still effectively cleared during mitotic exit when expressed from the *ACM1* promoter, with kinetics similar to wild-type Acm1-ProtA. There are several possible explanations for this surprising result. Although the Cdc20-specific D-box is absent from Acm1^NΔ52^ it is possible that removal of the N-terminal 52 amino acids makes the central D-box and/or KEN box that inhibit Cdh1 accessible to Cdc20. Cdh1 was also proposed to catalyze a slow ubiquitination and proteolysis of Acm1 [13] that, coupled with the termination of *ACM1* transcription, may contribute to clearance of endogenous protein. We confirmed that the slow turnover of Acm1^NΔ52^-ProtA in G1 cells is Cdh1-dependent (data not shown). Finally, there may be some difference in conditions present in an α-factor arrest compared to mitotic exit that affect the turnover rate of Acm1 by the APC-independent mechanism.

### Biological significance of Acm1 proteolytic mechanisms

Our results have confirmed the existence of two independent proteolytic mechanisms acting on Acm1. Acm1 is recognized by APC^Cdc20^ in anaphase as described previously [13] and degraded via the conventional ubiquitin pathway. However, APC^Cdc20^ is not sufficient to completely eliminate Acm1 and is not actually required for Acm1 levels to oscillate during the cell cycle (Figure 1). Consistent with this conclusion, mutation of the D-box in Acm1 that is recognized by Cdc20 had no noticeable effect on Acm1 stability or cell cycle expression profile [16], [23]. The APC-independent mechanism, which exhibits features of ubiquitin-independent proteolysis, is sufficient to establish Acm1’s cell cycle expression profile.

Why is APC^Cdc20^ insufficient for complete Acm1 degradation and why is it necessary to have two independent proteolytic mechanisms? One possibility is that two distinct cellular pools of Acm1 exist, one in the nucleus and one in the cytoplasm, and Cdc20 can only access nuclear Acm1. Although admittedly speculative, a fair amount of circumstantial evidence exists to support this possibility. Cdc20 is restricted primarily to the nucleus [49]. Cdh1, on the other hand, is restricted to the cytoplasm by Cdk phosphorylation until the end of anaphase [49]. Since Acm1 binds tightly to Cdh1 in cells arrested in late anaphase [11], it is likely that at least a portion of Acm1 remains cytoplasmic. Moreover, nuclear import of Acm1 is also negatively regulated by Cdk phosphorylation [13]. Since Acm1 levels are much higher than Cdh1 [11] a substantial pool of free Acm1 must exist that could be subject to rapid nucleocytoplasmic shuttling. When Cdc14 is first activated by the FEAR network in early anaphase, dephosphorylation of the pool of free Acm1 could trap it in the nucleus where it is recognized and targeted for degradation by APC^Cdc20^. The Cdh1-bound pool of Acm1 would be resistant to APC^Cdc20^ by virtue of its cytoplasmic localization. This pool of Acm1 could require the second ubiquitin-independent proteolytic mechanism activated when Cdc14, released by the mitotic exit network, reaches the cytoplasm and dephosphorylates both Cdh1 and Acm1. The complexity of Acm1 regulation in late mitosis strongly implies that timely and complete removal of Acm1 is essential for proper activation of APC^Cdh1^, completion of mitotic cyclin degradation, and exit from mitosis. Our observation of a growth delay caused by Acm1^NΔ52^ expression in *sic1Δ* cells supports this.

A related question is why APC^Cdc20^ action on Acm1 is required at all, especially considering that in the absence of APC activity Acm1 levels still cycle apparently normally (Figure 1). The N-terminal D box recognized by Cdc20 is highly conserved in Acm1 orthologs, suggesting that APC^Cdc20^-mediated degradation must be important. It may be that initial depletion of the abundance of free Acm1 by APC^Cdc20^ preconditions Cdh1 for rapid activation at the appropriate time in late mitosis, maximizing the efficiency, coordination, and robustness of the mitotic exit process.

Recently, Acm1 was proposed to act as a physiological buffer for Cdh1, precisely controlling Cdh1 activity in combination with inhibitory Cdk phosphorylation to allow proper multi-step assembly of a mitotic spindle [50]. In cells expressing a Cdh1 mutant lacking inhibitory Cdk phosphorylation sites, bipolar spindle assembly was ultra-sensitive to *ACM1* gene dosage. In this context, very fine control of Acm1 level by the phosphorylation-sensitive and constitutively active ubiquitin-independent proteolytic mechanism could precisely control Acm1 abundance at a level representing the maximal capacity for Cdk phosphorylation. Acm1 expressed above this level would begin to exceed the capacity of Cdk to maintain it in a phosphorylated state and result in rapid proteolysis of hypophosphorylated protein. This is consistent with our unpublished observations that overexpression of Acm1 from the *ADH* and *GAL1* promoters leads to only modest changes in steady-state level.

### Conclusion

The APC-independent proteolysis of Acm1 appears to represent a unique example of highly cell cycle-regulated proteasomal degradation independent of the canonical polyubiquitin targeting system. This mechanism also reflects the existence of alternative ways to establish cell cycle expression profiles other than via the SCF and APC E3 ligases. Our work reinforces the cooperative interplay of transcriptional and proteolytic control in establishing strict expression windows of cell cycle regulators during cell division. Future research on this topic should reveal if the unique cell cycle proteolysis of Acm1 represents a general mechanism complementing the ubiquitin proteasome system in governing cell cycle-dependent protein levels.

## Materials and Methods

### Strain and plasmid construction, and mutagenesis

All yeast strains used in this study, except those for screening ubiquitin system mutants, are listed in Table S2. Ubiquitin system mutant strains are listed in Table S1. All plasmids used in this study are listed in Table S3. Strain YKA468 was constructed by recombinational insertion of a PCR product containing a 3HA epitope tag with *klTRP1* selectable marker at the 3′ end of *CLB5* and a 9MYC tag with *HIS3MX6* selectable marker at the 3′ end of *PDS1* in the *dbf2-2* strain using plasmids pYM22 and pYM19 as templates as described [51]. Strain YKA469 was constructed by standard recombinational replacement of the *ACM1* coding sequence with a PCR-generated KanMX4 selectable marker. YKA859 was constructed from FM1175 by deleting *ACM1* using PCR-mediated integration of the KanMX4 marker and then inserting a 6HA:NatNT2 tag at the 3′ end of *KIP1* using pYM17 [51]. YKA404 was generated from Y7092 [52] by PCR-based deletion of *ACM1* with the Nat1 nourseothricin resistance cassette. E3 and E2 deletion strains for screening stability of untagged Acm1^5A^ were generated by crossing YKA404 to the appropriate MATa KanMX4 gene deletion strains from Open Biosystems as described [52]. Plasmid pHLP317 expressing 3HA-Fin1 from the *GAL1* promoter was constructed by subcloning the *BamHI*-*SalI* fragment from pESCW-Fin1-Myc into pESCLeu-3HA (both gifts from H. Charbonneau, Purdue University). The *acm1^K0^* mutant allele was created in pBluescript by site-directed mutagenesis using the QuikChange Multi kit (Stratagene) and, amplified by PCR, and subcloned into the *XhoI* sites of pHPL117 [11] to create pHLP328 or subcloned into the *SacII* and *XhoI* sites of p415GAL1 to create pHLP329. The one lysine codon in the 3HA sequence of pHLP329 was altered to an arginine codon to create pHLP330. The *3HA-acm1^K0^* alleles in pHLP328 and pHLP330 were confirmed by DNA sequencing. pHLP298 was constructed by sub-cloning the *3HA-ACM1^5A^* sequence from pHLP209 into p415GAL1. Plasmids for Acm1 stability experiments were constructed by first ligating the PCR-amplified ZZ domain of Protein-A into the HindIII and XhoI sites of p415GAL1 and then ligating the appropriate *ACM1* PCR fragments into the PstI and HindIII sites. All plasmid constructs were confirmed by DNA sequencing.

### Cell growth and cell cycle arrest

Standard yeast growth conditions and media were used. For G1 arrest, α-factor peptide (GenScript) was added to cultures at a final concentration of 5 µg/ml for *BAR1* or 50 µg/l for *bar1Δ* strains. For S phase arrest, solid hydroxyurea (Sigma Aldrich) was added directly to cultures at 10 mg/ml. For G2/M arrest, nocodazole (Sigma Aldrich) was added at a final concentration of 15 µg/ml from a 1.5 mg/ml stock in DMSO. For telophase arrest, strains harboring temperature-sensitive MEN mutants *cdc15-2* or *dbf2-2* were grown initially at 23°C and then shifted to 37°C. For all temperature sensitive strains, 23°C was used as permissive and 37°C as restrictive temperatures. For synchronous growth from G1 to telophase, α-factor treated *cdc15-2* or *dbf2-2* cells were released from arrest by extensive washing using a vacuum filtration device and resuspension in fresh medium pre-warmed to 37°C. Growth was continued at 37°C and samples were removed at the indicated times for analysis. Extracts of synchronized yBR135 and yBR159 cultures for analyzing Acm1 levels in the absence of APC activity [17] were generously provided by David Toczyski (U. California San Francisco). For metaphase block and release, FM1175 and its derivatives were grown in SD-Met-Leu media (50 mL) then transferred to YPD (120 mL) supplemented with 5 mM methionine and grown for 4 hours at 30°C. Cells were then released into 120 mL of selective media (SD-Met-Leu) following vacuum filtration onto a membrane disk (0.8 µm) and washing with 100 mL of SD-Met-Leu media. Cell cycle arrests were confirmed by microscopic analysis of cell morphology and flow cytometry. Flow cytometry analysis was performed exactly as described [11] using an Accuri C6 flow cytometer (BD Biosciences). Cell cycle stage in the synchronous growth experiments was determined by 4′,6-diamidino-2-phenylindole (DAPI) staining of formaldehyde-fixed cells and fluorescence microscopy to monitor nuclear division. Images were captured on an Olympus BX51 fluorescence microscope using Metamorph software (Molecular Devices, Inc.) and percentage of cells with segregated DNA masses quantified (minimum 100 cells per timepoint).

### Immunoblotting

The following antibodies were used. Monoclonal anti-HA 12CA5 and anti-Myc 9E10 were from Roche Applied Science (catalog #s11666606001 and 11667149001, respectively) and were used at concentrations of 0.5 µg/ml (1∶10,000 dilution) and 1 µg/ml (1∶5000 dilution), respectively. Rabbit anti-glucose-6-phosphate-dehydrogenase (G6PD) and rabbit anti-Protein A were from Sigma (catalog #sA9521 and P3775, respectively) and were used at concentrations of 3 ng/ml (1∶10,000 dilution) and 0.6 ng/ml (1∶50,000 dilution), respectively. Rabbit anti-Clb2, used at 40 ng/ml (1∶5,000 dilution) was from Santa Cruz Biotechnology (catalog #sc-9071). Horseradish peroxidase-conjugated donkey anti-rabbit and anti-mouse were from Jackson Immuno Research (catalog numbers 111-035-003 and 115-035-003, respectively) and were used at concentrations of 80 ng/ml each. Immunoblots were developed using ECL plus (GE Healthcare) or Luminata (Millipore) detection reagents.

For Acm1 polyclonal antibody production and purification, the complete *ACM1* open reading frame was cloned into pGEX6P-1 (GE Healthcare) for overexpression in *E. coli* as an N-terminal GST-fusion protein. The majority of GST-Acm1 is found in the insoluble fraction of bacterial cell extracts. Insoluble proteins were pelleted by centrifugation and resolubilized in 8 M urea, separated by SDS-PAGE and stained with Coomassie blue. The predominant band was GST-Acm1 and was excised and submitted to Pacific Immunology for polyclonal antibody production. Total Rabbit IgG was purified from serum using Protein A-agarose resin (Sigma Aldrich). Subsequently, anti-Acm1 antibodies were affinity purified using a GST-Acm1 affinity column generated by crosslinking recombinant GST-Acm1 to glutathione-agarose with disuccinimidyl suberate (Thermo Scientific). Acm1 antibody was eluted from the affinity column with 50 mM glycine pH 1.9 and immediately neutralized by addition of Tris-HCl pH 8.0 to 100 mM. Antibody specificity was tested by immunoblotting of yeast whole cell extracts using *acm1Δ* cells as a control. 1∶5,000 dilutions of the affinity purified antibody were used for all immunoblots.

Quantification of chemiluminescent immunoblots was performed using a Bio-Rad Laboratories ChemiDoc XRS+ digital imager and ImageLab software. Images obtained following incubation of blots with chemiluminescent reagent were analyzed using the Image Lab software. Signals were normalized to the G6PD load control signal and then to the 0 min timpoint.

### Protein stability assays

Measurements of protein stability were performed by *GAL1* promoter shutoff/cycloheximide chase assays as described [15]. Unless stated otherwise, expression was induced with 2% galactose and terminated by addition of 2% glucose and 0.5 mg/ml cycloheximide. For experiments in proteasome mutants (*cim3-1* and *pre1-1 pre2-2*), cells were first arrested with α-factor at 23°C and then protein expression induced for 30 min prior to shift to 37°C. For ubiquitin mutant overexpression experiments, actively growing cells were arrested with α-factor in early exponential phase (OD_600_ = ∼0.4) and then CuSO4 was added to 100 µM to induce overexpression of mutant or wild-type ubiquitin from the *CUP1* promoter. After 30 min 2% galactose was added to induce expression of the Acm1 or control proteins. To measure stability of endogenous Acm1, mid-exponential phase cells were treated only with 0.5 mg/ml cycloheximide to terminate expression. For MG-132 treatment, a *pdr5Δ* strain was used to maximize efficacy [53] and cultures were treated with 50 µM MG-132 (from 10 mM stock in DMSO) for 30 min prior to terminating expression.

For synchronized mitotic exit experiments, Acm1 or Acm1^NΔ52^-ProtA were expressed from the natural Acm1 promoter on CEN plasmids (pHLP117 or pHLP505, respectively) in a *P_MET3_-CDC20* background. Cultures were arrested in mitosis by Cdc20 depletion as described above and protein levels monitored over time by quantitative immunoblotting.

### Screening of ubiquitin system mutant strains

Yeast strains individually lacking all known non-essential E2 ubiquitin conjugating enzymes, E3 ubiquitin ligases, and proteasome components were obtained from the Open Biosystems yeast deletion library (Table S1). Relative levels and stability of wild-type Acm1 and the Acm1^5A^ mutant [15] were measured by immunoblotting as described above. For screening of essential E3 genes, strains from the Tet-repressible essential gene library (Table S1) containing pHLP209 expressing 3HA-Acm1^5A^ from the *ACM1* promoter were grown until mid-exponential phase and then treated with 2 µg/ml doxycycline for 2 hrs before processing for immunoblotting.

### In vivo APC inhibition assay

Inhibition of APC^Cdh1^ *in vivo* was measured exactly as described previously [11].

#### Spotting growth assay

Cultures of *sic1Δ* cells transformed with either p415ADH, pHLP361 (*P_ADH_*-*Acm1-ProtA*) or pHLP363 (*P_ADH_*-*acm1^NΔ52^-ProtA*) as well as isogenic wild-type and *cdh1Δ* strains transformed with p415ADH were grown to mid-exponential phase selective media, then washed and resuspended in sterile TE to OD_600_ of 1.0. Serial 10-fold dilutions were spotted onto SD-Leu agar plates and grown at 37°C for 2 days.

#### Growth rate measurements

Growth rates were measured by diluting exponentially growing cultures to an OD_600_ of 0.05 in selective media and growing in 96-well plates at 30°C with shaking in a BioTek Synergy 2 plate reader. Absorbance measurements at 600 nm were taken every 60 minutes.

#### Proteasome activity assays

Proteasomes were purified from yeast soluble whole cell extracts by IgG-agarose (Sigma-Aldrich) affinity chromatography using a protocol described previously [54]. For 26S proteasomes, a strain expressing an *RPN11-TAP* fusion was used and for 20S proteasomes a strain expressing a *PRE1-TEVProA* fusion was used. To assay general activity of proteasome preparations, the fluorogenic substrate Suc-LLVY-AMC (Peptides International, Inc.) was used as described [55] at 100 µM in reaction buffer (50 mM Tris-HCl pH 7.4, 5 mM MgCl_2_, 10% glycerol, and 1 mM ATP). Fluorescence of the hydrolyzed AMC moiety was measured at excitation and emission wavelengths of 380 and 440 nm, respectively. Specific activities of purified 26S proteasome and 20S proteasomes were measured as described [55]. Values for the 20S proteasome were comparable to that observed in other studies [55]. We did not find reports of specific activities for the budding yeast 26S proteasome on the Suc-LLVY-AMC substrate under similar conditions.

GST-Acm1 and 6His-Acm1 were purified from *E. coli* as described previously [23], [56]. The GST tag was cleaved off Acm1 with PreScission (3C) protease (GE Life Sciences). To assay degradation of purified GST-Acm1 by the 26S proteasome preparation, 200 µg of GST-Acm1 and 500 µg proteasome were mixed in 500 µl of reaction buffer with 100 mM NaCl at 30°C and samples were collected over time and analyzed by western blot using α-Acm1 antibody. Degradation of 6His-Acm1 by 26S and 20S proteasomes was assayed similarly for 30 min at 30°C. Free Acm1 was generated by pre-treatment of GST-Acm1 with 3C protease before mixing with 26S proteasome and proceeding as above. 5 µM epoxomicin (Peptides International, Inc.) was used where indicated to specifically inhibit the proteasome.

## Supporting Information

Figure S1 **Screening of non-essential E2 conjugases and E3 ligases for effects on 3HA-Acm1^5A^ expression level.**
(PDF)

Figure S2 **Measurements of HA-Acm1^5A^ stability in selected strains lacking non-essential E2 conjugases and E3 ligases.**
(PDF)

Figure S3 **Screening of non-essential E2 conjugases and known and putative E3 ligases for effects on Acm1^5A^ stability.**
(PDF)

Figure S4 **Acm1 is not degraded by purified 20S or 26S proteasomes.**
(PDF)

Table S1 **Yeast deletion strains screened for effects on Acm1 stabilization.**
(PDF)

Table S2 **Yeast strains used in this study.**
(PDF)

Table S3 **Yeast plasmids used in this study.**
(PDF)
