# Supplementary material for: Timely Activation of Budding Yeast APCCdh1 Involves Degradation of Its Inhibitor, Acm1, by an Unconventional Proteolytic Mechanism
Source: PLoS One. 2014 Jul 29;9(7):e103517. doi: 10.1371/journal.pone.0103517 (PMC4114781; doi:10.1371/journal.pone.0103517)
Supplement: Figure S1 — Screening of non-essential E2 conjugases and E3 ligases for effects on 3HA-Acm15A expression level. (PDF) [file pone.0103517.s001.pdf]

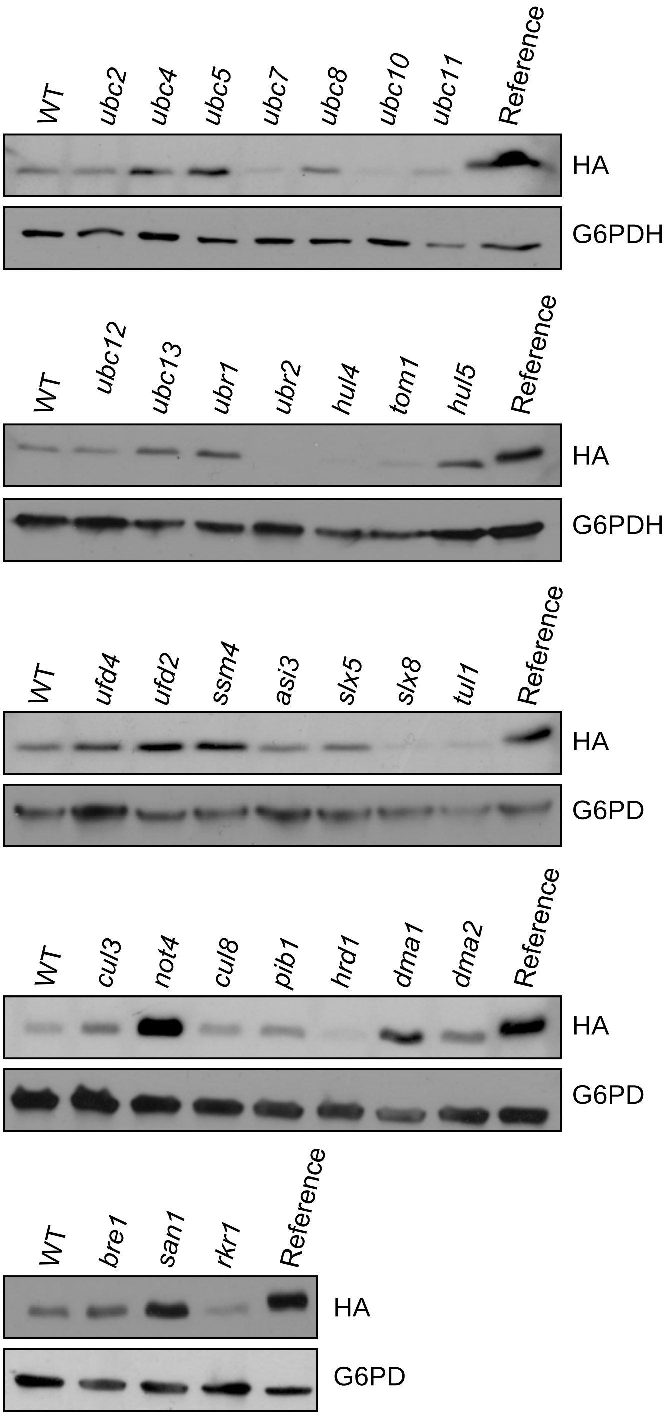

Figure S1. Screening of non-essential E2 conjugases and E3 ligases for effects on 3HA-Acm1^{5A} expression level. 3HA-Acm1^{5A} was expressed from the natural *ACM1* promoter on a centromeric plasmid in BY4741 (WT) and deletion strains lacking the genes indicated above each lane. The steady state level of 3HA-Acm1^{5A} in asynchronous log phase cultures of each strain was compared to that of wild-type 3HA-Acm1 (labeled "Reference") expressed from the same plasmid in BY4741 by immunoblotting with anti-HA antibody. G6PDH is a loading control.
