# Supplementary material for: Timely Activation of Budding Yeast APCCdh1 Involves Degradation of Its Inhibitor, Acm1, by an Unconventional Proteolytic Mechanism
Source: PLoS One. 2014 Jul 29;9(7):e103517. doi: 10.1371/journal.pone.0103517 (PMC4114781; doi:10.1371/journal.pone.0103517)
Supplement: Figure S2 — Measurements of HA-Acm15A stability in selected strains lacking non-essential E2 conjugases and E3 ligases. (PDF) [file pone.0103517.s002.pdf]

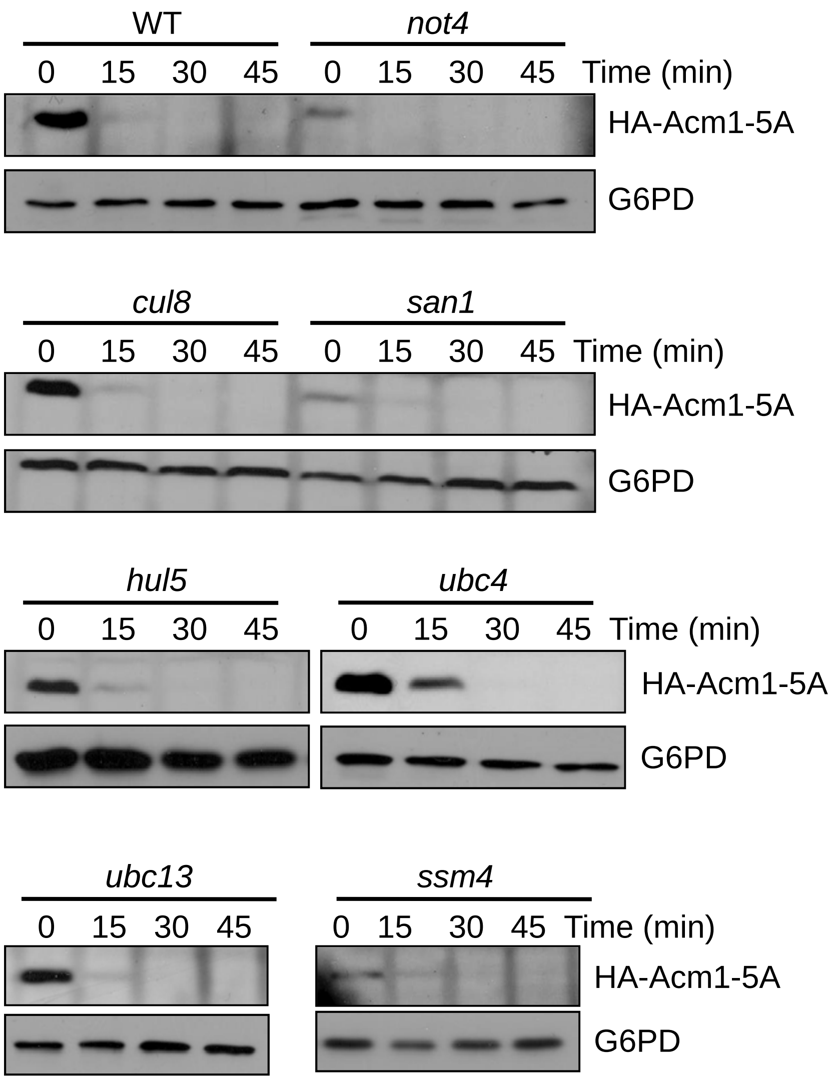

Figure S2. Measurements of HA-Acm1^{5A} stability in selected strains lacking non-essential E2 conjugases and E3 ligases. Stability of HA-Acm1^{5A} was monitored by *GAL1* promoter stability assay as described in Materials and Methods in asynchronous mid-log phase cultures of the indicated gene deletion strains harboring pHLP110. Anti-HA immunoblot profiles were compared to those from MG-132 treated cells and conditional proteasome mutant strains (not shown, see Figure 2).
