# Supplementary material for: Timely Activation of Budding Yeast APCCdh1 Involves Degradation of Its Inhibitor, Acm1, by an Unconventional Proteolytic Mechanism
Source: PLoS One. 2014 Jul 29;9(7):e103517. doi: 10.1371/journal.pone.0103517 (PMC4114781; doi:10.1371/journal.pone.0103517)
Supplement: Figure S3 — Screening of non-essential E2 conjugases and known and putative E3 ligases for effects on Acm15A stability. (PDF) [file pone.0103517.s003.pdf]

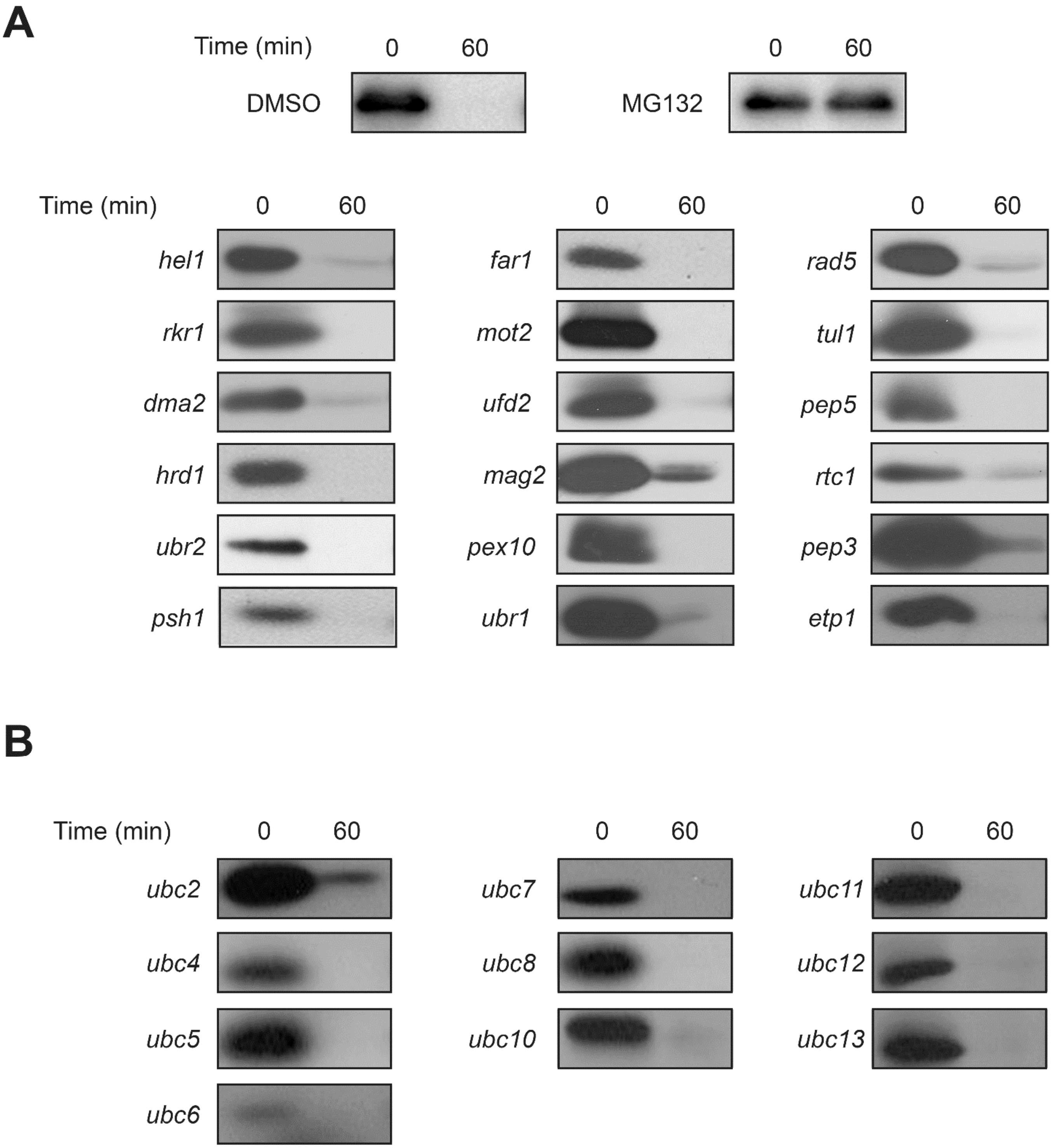

Figure S3. Screening of non-essential E2 conjugases and known and putative E3 ligases for effects on Acm1^{5A} stability. Experiments performed as in Figure S2 but using an untagged Acm1^{5A} variant expressed from P_{GAL1} in pHLP392 in an *acm1Δ* background. Only timepoints 0 and 60 minutes following glucose and cycloheximide addition were compared. MG-132 treatment was used as a positive control for Acm1 stabilization. Anti-Acm1 antibody was used to detect the Acm1^{5A}. Only representative E3 deletion strains are shown in panel A, but all strains listed in Table S1 were analyzed with similar results.
