# Supplementary material for: Timely Activation of Budding Yeast APCCdh1 Involves Degradation of Its Inhibitor, Acm1, by an Unconventional Proteolytic Mechanism
Source: PLoS One. 2014 Jul 29;9(7):e103517. doi: 10.1371/journal.pone.0103517 (PMC4114781; doi:10.1371/journal.pone.0103517)
Supplement: Figure S4 — Acm1 is not degraded by purified 20S or 26S proteasomes. (PDF) [file pone.0103517.s004.pdf]

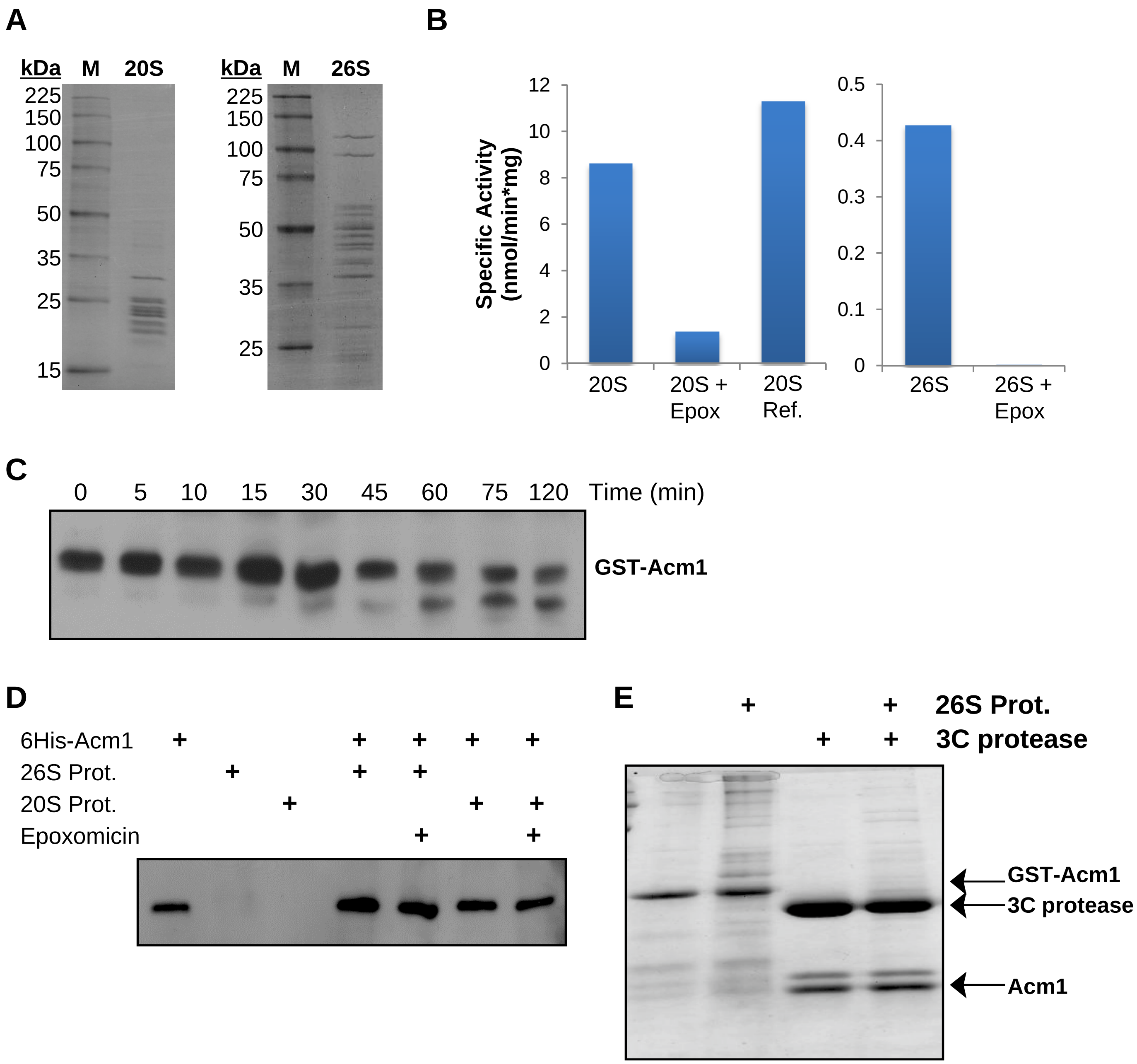

Figure S4. Acm1 is not degraded by purified 20S or 26S proteasomes. A) SDS-PAGE analysis of purified 20S (left) and 26S (right) proteasome preparations visualized with Coomassie blue. M, molecular weight markers. Components of the proteasomes were confirmed by mass spectrometry. B) Proteasome activities were measured using the fluorogenic substrate, Suc-LLVY-AMC. The proteasome inhibitor epoxomycin (Epox) was used to demonstrate specificity. Similar results were observed with MG-132 (not shown). A standard curve of free AMC was used to convert fluorescence signal to moles of product formed. 20S Ref is the specific activity value reported previously for budding yeast 20S proteasome in reference 55 from the main text. C) Immunoblot of recombinant purified GST-Acm1 incubated with purified 26S proteasome over time. D) Immunoblot of recombinant 6His-Acm1 alone and after incubation with purified proteasome (20S or 26S) with and without the inhibitor epoxomicin. E) GST-Acm1 and free Acm1 generated by treatment of GST-Acm1 with 3C protease were incubated with purified 26S proteasome and analyzed by SDS-PAGE and Coomassie blue staining.
