# Supplementary material for: Timely Activation of Budding Yeast APCCdh1 Involves Degradation of Its Inhibitor, Acm1, by an Unconventional Proteolytic Mechanism
Source: PLoS One. 2014 Jul 29;9(7):e103517. doi: 10.1371/journal.pone.0103517 (PMC4114781; doi:10.1371/journal.pone.0103517)
Supplement: Table S1 — Yeast deletion strains screened for effects on Acm1 stabilization. (PDF) [file pone.0103517.s005.pdf]

Table S1. Yeast deletion strains screened for effects on Acm1 stabilization.

Non-essential E3s		Non-essential E2s	Essential E3s
ASI3	*PEX12*	***UBC2***	*RSP5*
ASR1	***PIB1***	***UBC4***	*PRP19*
BRE1	*PSH1*	***UBC5***	*SCF**
CUL3	*RAD5*	*UBC6*	*APC**
CUL8	*RAD16*	***UBC7***	
DMA1	*RAD18*	***UBC8***	
DMA2	*RCO1*	***UBC10***	
ETP1	***RKR1***	***UBC11***	
FAP1	*RTC1*	***UBC12***	
FAR1	*SAN1*	***UBC13***	
HEL1	***SLX5***		
HEL2	***SLX8***		
HRD1	*SNT2*		
HUL4	***SSM4***		
HUL5	***TOM1***		
IRC20	*TUL1*		
ITT1	***UBR1***		
MAG2	***UBR2***		
MOT2(NOT4)	***UFD2***		
NFI1	***UFD4***		
PEP3	*ULS1*		
PEP5	*VPS8*		
PEX2	*YBR062C*		
PEX10			

For all non-essential genes, deletion strains were obtained from the Open Biosystems gene deletion library and crossed to YKA404 to generate combined deletions with *acm1Δ::*KanMX4.

For essential genes RSP5 and PRP19, tetracycline-repressible strains were obtained from the Open Biosystems Tet-promoter collection.

*Effects of SCF on Acm1 stability were tested using conditional *cdc34* and *cdc53* mutant strains (not shown and [32]). No effect was found. Lack of dependence on APC can be found in Figure 1 and [31].

Strains in bold were tested in both Figure S1 and Figure S3 screens. Non-essential E3 and E2 strains not in bold were only tested in the Figure S3 screen.
