# Supplementary material for: Timely Activation of Budding Yeast APCCdh1 Involves Degradation of Its Inhibitor, Acm1, by an Unconventional Proteolytic Mechanism
Source: PLoS One. 2014 Jul 29;9(7):e103517. doi: 10.1371/journal.pone.0103517 (PMC4114781; doi:10.1371/journal.pone.0103517)
Supplement: Table S2 — Yeast strains used in this study. (PDF) [file pone.0103517.s006.pdf]

Table S2. Yeast strains used in this study.

Strain	Genotype	Source
BY4741	MATa *his3Δ1 leu2Δ0 met15Δ0 ura3Δ0*	Open Biosystems
W303	MATa *ade2-1 his3-11,15 leu2-3,112 trp1-1 ura3-1 can1-100*	
doa4□	(BY4741) MATa *doa4*::KanMX4	Open Biosystems
MHY753	MATa *his3-Δ200 leu2D1 ura3-52 lys2-801 trp1-63 ade2-101*	[19]
MHY754	MATa *his3-Δ200 leu2D1 ura3-52 lys2-801 trp1-63 ade2-101 cim3-1*	[19]
YKA247	(W303) *bar1*::URA3 *acm1*::KanMX4	[11]
YKA404	MATα *acm1::*Nat1 *can1::STE2pr-his5 lyp1Δ his3Δ1 leu2Δ0 ura3Δ0 met15Δ0*	This study
YKA407	(BY4741) MATa *bar1*::hisG *acm1*::KanMX4 *pdr5*::*URA3*	[15]
YWO0607	MATa *ura3 leu2-3,112 his3-11,15* Can^S Gal+	Dieter H. Wolf
YWO0612	MATa *ura3 leu2-3,112 his3-11,15* Can^S Gal+ *pre1-1 pre2*-2	Dieter H. Wolf
YKA468	MATa *ura3Δ leu2Δ ade2Δ his3Δ trp1Δ dbf2-2 CLB5-3HA:TRP1 PDS1-9MYC:HIS3*	This study
YKA469	(W303) MATa *cdc15-2 GFP-TUB1:URA3 acm1::KanMX4*	This study
yBR135	(W303) MATa *pds1::LEU2 clb5::HIS3 trp1-1::SIC1:*$TRP1^{10x}$	[17]
yBR159	(W303) MATa *pds1::LEU2 clb5::HIS3 trp1-1::SIC1:*$TRP1^{10x}$ *apc2Δ apc11Δ cdc20Δ cdh1Δ*	[17]
RJD3268	(W303) MATa *uba1*::KanMX pRS313-*UBA1*	Ray Deshaies
RJD3269	(W303) MATa *uba1*::KanMX pRS313-*uba1-204*	Ray Deshaies
FM1175	(W303) MATa P_{MET3}*-CDC20-HA::TRP1*	Foong May Yeong
YKA859	(FM1175) *acm1*::KanMX4 *KIP1-6HA*:NatNT2	This study
*sic1*Δ	(BY4741) MATa *sic1*::KanMX4	Open Biosystems
cdh1Δ	(BY4741) MATa *cdh1*::KanMX4	Open Biosystems
RPN11-TAP	(BY4741) MATa *RPN11-TAP:HIS3MX*	Open Biosystems
SDL135	MATa *lys2-801 leu2-3,112 ura3-52 his3-Δ200 trp1-1 pre1::PRE1-TEVProA:HIS3*	Daniel Finley
