# Supplementary material for: Timely Activation of Budding Yeast APCCdh1 Involves Degradation of Its Inhibitor, Acm1, by an Unconventional Proteolytic Mechanism
Source: PLoS One. 2014 Jul 29;9(7):e103517. doi: 10.1371/journal.pone.0103517 (PMC4114781; doi:10.1371/journal.pone.0103517)
Supplement: Table S3 — Yeast plasmids used in this study. (PDF) [file pone.0103517.s007.pdf]

Table S3. Yeast plasmids used in this study.

Name	Expressed Protein	Backbone	Marker	Origin	Promoter	Source
LHP306	Ub-K7R	YEp352	*URA3*	2μm	*CUP1*	*[22]*
LHP308	Ubiquitin	YEp352	*URA3*	2μm	*CUP1*	*[22]*
pESCTrp-Fin1	Fin1-Myc	pESC-Trp	*TRP1*	2μm	*GAL1*	*[15]*
pHLP110	HA-Acm1^{5A}	p415GAL1	*LEU2*	CEN/ARS	*GAL1*	*[15]*
pHLP117	3HA-Acm1	p415ADH	*LEU2*	CEN/ARS	*ACM1*	*[11]*
pHLP123	HA-Acm1^{ken}	p415GAL1	*LEU2*	CEN/ARS	*GAL1*	*[23]*
pHLP209	3HA-Acm1^{5A}	p415ADH	*LEU2*	CEN/ARS	*ACM1*	*[15]*
pHLP212	3HA-Acm1	p415GAL1	*LEU2*	CEN/ARS	*GAL1*	*[15]*
pHLP231	3FLAG-Cdh1	pNC219	*TRP1*	CEN/ARS	*GAL1*	*[15]*
pHLP298	3HA-Acm1^{5A}	p415GAL1	*LEU2*	CEN/ARS	*GAL1*	This study
pHLP309	Clb2	P415GALL	*LEU2*	CEN/ARS	*GALL*	This study
pHLP317	Fin1-3HA	pESC-Leu	*LEU2*	2μm	*GAL1*	This study
pHLP328	3HA-Acm1^{K0}	p415ADH	*LEU2*	CEN/ARS	*ACM1*	This study
pHLP330	3HA-Acm1^{K0}	p415GAL1	*LEU2*	CEN/ARS	*GAL1*	This study
pHLP391	Acm1	P415GAL1	*LEU2*	CEN/ARS	*GAL1*	This study
pHLP392	Acm1^{5A}	p415GAL1	*LEU2*	CEN/ARS	*GAL1*	This study
pHLP397	Acm1-ProtA	p415GAL1	*LEU2*	CEN/ARS	*GAL1*	This study
pHLP399	Acm1$^{N\Delta42}$-ProtA	p415GAL1	*LEU2*	CEN/ARS	*GAL1*	This study
pHLP400	Acm1$^{N\Delta52}$-ProtA	p415GAL1	*LEU2*	CEN/ARS	*GAL1*	This study
pHLP401	Acm1$^{N\Delta60}$-ProtA	p415GAL1	*LEU2*	CEN/ARS	*GAL1*	This study
pHLP402	Acm1$^{N\Delta72}$-ProtA	p415GAL1	*LEU2*	CEN/ARS	*GAL1*	This study
pHLP403	Acm1$^{N\Delta80}$-ProtA	p415GAL1	*LEU2*	CEN/ARS	*GAL1*	This study
pHLP413	Acm1$^{1-18}$-ProtA	p415GAL1	*LEU2*	CEN/ARS	*GAL1*	This study
pHLP414	Acm1$^{13-30}$-ProtA	p415GAL1	*LEU2*	CEN/ARS	*GAL1*	This study
pHLP415	Acm1$^{25-42}$-ProtA	p415GAL1	*LEU2*	CEN/ARS	*GAL1*	This study
pHLP416	Acm1$^{37-54}$-ProtA	p415GAL1	*LEU2*	CEN/ARS	*GAL1*	This study
pHLP417	Acm1$^{49-66}$-ProtA	p415GAL1	*LEU2*	CEN/ARS	*GAL1*	This study
pHLP503	Acm1$^{1-52}$-ProtA	p415GAL1	*LEU2*	CEN/ARS	*GAL1*	This study
pHLP504	Acm1$^{12-52}$-ProtA	p415GAL1	*LEU2*	CEN/ARS	*GAL1*	This study
pHLP505	Acm1$^{N\Delta52}$-ProtA	p415GAL1	*LEU2*	CEN/ARS	*ACM1*	This study
pHLP361	Acm1-ProtA	p415ADH	*LEU2*	CEN/ARS	*ADH1*	This study
pHLP363	Acm1$^{N\Delta52}$-ProtA	p415ADH	*LEU2*	CEN/ARS	*ADH1*	This study
